# Increased Neuronal Differentiation of Neural Progenitor Cells Derived from Phosphovimentin-Deficient Mice

**DOI:** 10.1007/s12035-017-0759-0

**Published:** 2017-09-27

**Authors:** Meng Chen, Till B. Puschmann, Pavel Marasek, Masaki Inagaki, Marcela Pekna, Ulrika Wilhelmsson, Milos Pekny

**Affiliations:** 1 Laboratory of Astrocyte Biology and CNS Regeneration, Center for Brain Repair and Rehabilitation, Department of Clinical Neuroscience, Institute of Neuroscience and Physiology, Sahlgrenska Academy at the University of Gothenburg, Box 440, 40530 Gothenburg, Sweden; 2 Department of Physiology, Mie University Graduate School of Medicine, Mie, Japan; 3 Laboratory of Regenerative Neuroimmunology, Center for Brain Repair and Rehabilitation, Department of Clinical Neuroscience, Institute of Neuroscience and Physiology, Sahlgrenska Academy at the University of Gothenburg, Gothenburg, Sweden; 4 Florey Institute of Neuroscience and Mental Health, Parkville, VIC Australia; 5 University of Newcastle, Newcastle, NSW Australia

**Keywords:** Intermediate filaments, Nanofilaments, Vimentin, GFAP, Astrocytes, Neural stem/progenitor cells, Bioactive3D culture system

## Abstract

Vimentin is an intermediate filament (also known as nanofilament) protein expressed in several cell types of the central nervous system, including astrocytes and neural stem/progenitor cells. Mutation of the vimentin serine sites that are phosphorylated during mitosis (*VIM* ^*SA/SA*^) leads to cytokinetic failures in fibroblasts and lens epithelial cells, resulting in chromosomal instability and increased expression of cell senescence markers. In this study, we investigated morphology, proliferative capacity, and motility of *VIM* ^*SA/SA*^ astrocytes, and their effect on the differentiation of neural stem/progenitor cells. *VIM* ^*SA/SA*^ astrocytes expressed less vimentin and more GFAP but showed a well-developed intermediate filament network, exhibited normal cell morphology, proliferation, and motility in an in vitro wound closing assay. Interestingly, we found a two- to fourfold increased neuronal differentiation of *VIM* ^*SA/SA*^ neurosphere cells, both in a standard 2D and in Bioactive3D cell culture systems, and determined that this effect was neurosphere cell autonomous and not dependent on cocultured astrocytes. Using BrdU in vivo labeling to assess neural stem/progenitor cell proliferation and differentiation in the hippocampus of adult mice, one of the two major adult neurogenic regions, we found a modest increase (by 8%) in the fraction of newly born and surviving neurons. Thus, mutation of the serine sites phosphorylated in vimentin during mitosis alters intermediate filament protein expression but has no effect on astrocyte morphology or proliferation, and leads to increased neuronal differentiation of neural progenitor cells.

## Introduction

The intermediate filament system (known also as nanofilament system) of astrocytes is a dynamic integrator of cellular functions under physiological conditions and plays an important role in times of cellular stress as well as in the subsequent regenerative processes [1–6]. Whereas the upregulation of intermediate filament proteins in astrocytes is important for the confinement of the lesion area in brain injury, ischemic stroke, retinal ischemia, or spinal cord injury, it inhibits some of the regenerative processes later on [4, 5]. We and others previously demonstrated that mice carrying null mutations in genes encoding glial fibrillary acidic protein (GFAP) and vimentin (*GFAP* ^*−/−*^ *Vim* ^*−/−*^ mice) have astrocytes devoid of astrocyte intermediate filaments [7, 8] and exhibit better posttraumatic regeneration of neuronal synapses and axons [9, 10], improved functional recovery after spinal cord injury [11], reduced photoreceptor degeneration in the retinal detachment model [12], and reduced pathological neovascularization in oxygen-induced retinopathy [13]. We also demonstrated that in *GFAP* ^*−/−*^ *Vim* ^*−/−*^ mice, retinal grafts can better integrate [14], differentiation of transplanted neural stem cells into neurons and astrocytes is enhanced [15], and hippocampal neurogenesis is increased in naïve mice [16], after neonatal hypoxic-ischemic injury [17], or after neurotrauma [16]. The astrocyte intermediate filament system is important for the ability of astrocytes to cope with conditions associated with cellular stress, such as that induced by ischemia reperfusion [18–20]. We have shown that the astrocyte intermediate filament system regulates Notch signaling from astrocytes to neural stem/progenitor cells, a mechanism that inhibits differentiation of neural progenitors into neurons, astrocytes, or oligodendrocytes in the adult brain [16, 21]. Thus, in a variety of injury models, the benefits of reactive gliosis in the acute stage of central nervous system injury is balanced against restricted regenerative potential at the later stage, and hence modulation of reactive gliosis targeting the intermediate filament system might lead to enhanced recovery after central nervous system injury.

The highly dynamic assembly and disassembly of intermediate filaments is essential for the function of the intermediate filament system [22–24]. Intermediate filament phosphorylation is a key regulator of intermediate filament dynamics and is crucial for the organization of the intermediate filament network and the subcellular distribution of intermediate filament proteins [25, 26]. The intermediate filament disassembly, regulated by phosphorylation of serine/threonine residues in the amino-terminal head domain of intermediate filament proteins [24, 27], was reported to be essential for the efficient separation of the two daughter cells during mitosis [28–32]. In various cell types, including astrocytes, some of the key vimentin phosphorylation sites and their respective protein kinases have been identified [28–30, 33–41]. Phosphovimentin-deficient mice (*VIM* ^*SA/SA*^ mice), i.e., mice expressing vimentin in which all the serine sites that are phosphorylated during mitosis were substituted by alanine residues, show cytokinetic failures in fibroblasts and lens epithelial cells resulting in aneuploidy, chromosomal instability, and increased expression of cell senescence markers [42]. *VIM* ^*SA/SA*^ mice exhibit a phenotype of pre-mature aging, including cataract development in lens, delayed skin wound healing, and subcutaneous fat loss in old age [42, 43]. Here, we investigated whether the vimentin phosphorylation deficit in *VIM* ^*SA/SA*^ mice alters astrocyte morphology, proliferative capacity, and motility, and whether the phosphovimentin-deficient astrocyte niche affects neuronal differentiation of neural progenitor cells in vitro and neurogenesis in vivo.

## Experimental Procedures

### Animals

In *Vim* ^*SA/SA*^ mice, the serine residues in the vimentin head domain identified as phosphorylation sites during mitosis (Ser-6, Ser-24, Ser-38, Ser-46, Ser-55, Ser-64, Ser-65, Ser-71, Ser-72, Ser-82, and Ser-86) were replaced by alanine [42]. The *Vim* ^*SA*^ mutation was on C57Bl/6 genetic background, the colony was maintained as heterozygotes, and the experimental groups were genotyped by PCR. All mice were housed in standard cages in a barrier animal facility and had free access to food and water. All the experiments were conducted according to protocols approved by the Ethics Committee of the University of Gothenburg (Dnr. 247–2014).

### Astrocyte Cultures

Postnatal day 0.5–2.5 mouse cortical tissue was dissected in cold Dulbecco’s phosphate-buffered saline (DPBS) (Thermo Fisher Scientific), cut into small pieces, incubated in 0.05% trypsin-ethylenediaminetetraacetic acid (EDTA) solution (Thermo Fisher Scientific) at 37 °C for 10 min, and mechanically dissociated into a single cell suspension. Single cell suspension isolated from each mouse brain were seeded in a poly-d-lysine-coated (10 μg/mL; Sigma-Aldrich) T75 culture flask (Sarstedt) in Dulbecco’s minimal essential medium (DMEM) (Thermo Fisher Scientific) supplemented with 1% Pen/Strep (Thermo Fisher Scientific), 1% l-glutamine (Thermo Fisher Scientific), and 10% heat-inactivated fetal calf serum (FCS; HyClone/Thermo Fisher Scientific). The contamination of non-astrocyte cells in these cultures was minimalized as previously described [19, 44]. For astrocyte proliferation assay, 10,500 cells/cm^2^ were seeded in poly-d-lysine-coated 6-well culture plates. For scratch wound live imaging recording, 12,500 cells/cm^2^ were seeded in poly-d-lysine-coated 12-well culture plates. For intermediate filament bundle imaging and cell size/polynucleation assessment, primary astrocytes were detached (trypsinized) by incubating with 0.25% trypsin-EDTA solution (Thermo Fisher Scientific) at 37 °C for 10 min and reseeded on poly-d-lysine-coated coverslips placed in 24-well culture plates (TPP), at a density of 30,000 cells/cm^2^.

### Astrocyte Proliferation Assay and Scratch Wound Healing Assay

For astrocyte proliferation assay, primary astrocytes cultured in 6-well culture plates were collected on 4, 8, 12, 16, 20, and 24 days in vitro (DIV), trypsinized, and resuspended in astrocyte medium for cell counting. The cell number per milliliter medium was determined by a Countess automated cell counter (Thermo Fisher Scientific). For astrocyte wound healing assay, an approximate 800 μm wide wound was made by scratching with a 1-mL pipette tip on 14-day-old primary astrocytes cultured in a 12-well culture plate. The plate was then placed on a Leica DMI 6000B microscope connected to an incubator chamber supplied with humid atmosphere of 37 °C and 5% CO_2_. Images were taken every 12 h automatically for a total of 132 h.

### Neurosphere Cultures

Postnatal day 2.5–3.5 mouse forebrain was dissected in cold DPBS (Thermo Fisher Scientific), cut into small pieces, incubated in TrypLE solution (Thermo Fisher Scientific) at 37 °C for 10 min, and mechanically dissociated into a single cell suspension. The dissociated cells were plated in neurosphere medium composed of Neurobasal medium (Thermo Fisher Scientific), supplemented with 1% Pen/Strep (Thermo Fisher Scientific), 1% l-glutamine (Thermo Fisher Scientific), 2% B27 (Thermo Fisher Scientific), 20 ng/mL bFGF (Thermo Fisher Scientific), 20 ng/mL EGF (Thermo Fisher Scientific), and 1 U/mL Heparin (Sigma-Aldrich). Dissociated brain cells were plated at a density of 8000 cells/cm^2^ in T25 cell suspension culture flasks (Sarstedt; for neurosphere expansion) or 12,500 cells/cm^2^ in 12-well suspension culture plates (Greiner Bio One; for neurosphere quantification) to form neurospheres. Neurospheres were passaged on 7 DIV by dissociation of neurosphere cells with TrypLE solution (Thermo Fisher Scientific) into a single cell suspension, which was then replated. To quantify secondary and quaternary neurospheres, primary and tertiary neurospheres, respectively, were dissociated and plated at a density of 5000 cells/cm^2^ in 48-well cell suspension culture plates (Greiner Bio One).

### Neurosphere Differentiation

On 7 DIV, primary neurospheres were collected, dissociated by TrypLE, and allowed to differentiate in differentiation medium on poly-l-ornithine- (0.01 mg/mL; Sigma-Aldrich) and laminin-coated (5 μg/mL; Thermo Fisher Scientific) 24-well culture plates (TPP) or on poly-l-ornithine- and laminin-coated Bioactive3D nanofiber scaffolds (3Dtro) placed in 24-well culture plates. The differentiation medium was composed of Neurobasal medium (Thermo Fisher Scientific) supplemented with 1% Pen/Strep (Thermo Fisher Scientific), 1% l-glutamine (Thermo Fisher Scientific), 2% B27 (Thermo Fisher Scientific), and 1% heat-inactivated fetal calf serum (HyClone/Thermo Fisher Scientific). Differentiated cells were cultured for 5 days before examination. For immunocytochemical analysis, neurospheres were allowed to adhere on a poly-d-lysine-coated 24-well culture plate, and cultured for 24 h before examination.

### Astrocyte–Neurosphere Cocultures

At confluency (6–7 DIV), primary astrocytes were trypsinized and plated in poly-l-ornithine- and laminin-coated 24-well culture plates or Bioactive3D nanofiber scaffolds placed in 24-well culture plates. Twenty-four hours later, the culture medium was changed to neurosphere differentiation medium. When confluent (2–3 days), dissociated primary neurosphere cells were plated on astrocytes. To label the neurosphere cells, 0.5 μM 5-bromo-2′-deoxyuridine (BrdU) was added into the neurosphere culture 48 h before dissociation. The neurosphere cells were allowed to differentiate in the coculture for 5 days before examination.

### Immunocytochemistry

Cells were washed in DPBS and fixed in 4% paraformaldehyde (PFA; Sigma-Aldrich), followed by blocking unspecific binding sites using blocking buffer. The blocking buffer consisted of DPBS supplemented with 2% donkey serum (Jackson Immunoresearch), 2% goat serum (Jackson Immunoresearch), and 0.1% Triton-X 100 (Sigma-Aldrich). The cells were then incubated with primary antibodies at 4 °C overnight. For detection of BrdU, cells were first treated with 2 M HCl at 37 °C for 10 min. Proteins of interest were detected using the following primary antibodies: rabbit anti-nestin (1:1500; Covance), rabbit anti-GFAP (1:1500; Dako), chicken anti-vimentin (1:1500; Biolegend), mouse anti-βIII-tubulin (1:1500; Covance), mouse anti-MAP2 (1:1500; Sigma-Aldrich), and rat anti-BrdU (1:200; Serotec). Fluorophore-conjugated secondary antibodies were used at a dilution of 1:2000 and incubated with the cells for 1.5 h at room temperature (RT). Secondary antibodies used were as follows (all from Thermo Fisher Scientific): Alexa 594-conjugated goat anti-mouse, Alexa 488-conjugated donkey anti-mouse, Alexa 555-conjugated donkey anti-rabbit, Alexa 488-conjugated donkey anti-rabbit, Alexa 488-conjugated donkey anti-rat, Alexa 647-conjugated goat anti-chicken, and Alexa 647-conjugated goat anti-rat. Cell nuclei were visualized by DAPI (Sigma-Aldrich). Fluorescence-labeled cell cultures were imaged and analyzed using either a Leica DMI 6000B microscope (Leica) or a LSM 700 confocal microscope (Zeiss). Only βIII-tubulin^pos^ cells negative for GFAP were regarded as neurons and only GFAP^pos^ cells negative for βIII-tubulin were regarded as astrocytes. The images were analyzed by ImageJ software.

For astrocyte cell size and polynucleation assessment, the CellTracker dye (Thermo Fisher Scientific) was diluted 50× with PBS and incubated with cells for 20 min at RT. Nuclei of these cells were counterstained with TO-PRO-3 dye (Thermo Fisher Scientific). The coverslips were scanned with ScanR highcontent microscope (Olympus) and analyzed using ScanR Analysis software. The software was used for determining the cell size and counting total number of cells, polynucleated cells were counted manually.

### Protein Extraction and Western Blot Analysis

Protein extraction protocols were previously described [45]. In brief, total protein from astrocytes or differentiated neurosphere cells was harvested by adding protein lysis buffer to the cell culture plates for 2D or by submerging the nanofiber scaffolds in an Eppendorf tube containing lysis buffer for Bioactive3D cell cultures. Neurospheres were collected in a 15-mL tube and centrifuged at 300×*g* for 5 min, the culture medium was removed, and neurospheres washed with DPBS and resuspended in a protein lysis buffer. The protein lysis buffer was composed of 20 mM Tris-HCL (pH 7.5), 150 mM NaCl, 1 mM EDTA, 1% *v*/v Triton-X 100, one tablet of protease inhibitor (Roche)/10 mL lysis buffer, and one tablet of phosphatase inhibitor (Roche)/10 mL lysis buffer. The protein lysates were sonicated for 30 s at 14 amplitude microns followed by protein concentration measurement by using Bio-Rad DC protein assay (Bio-Rad).

Sodium dodecyl sulfate polyacrylamide gel electrophoresis (SDS-PAGE) was performed using Any-kD polyacrylamide gels (Bio-Rad) and Tris-glycine running buffer (Bio-Rad). A total amount of 15 μg of protein lysate per lane was loaded. Separated proteins were blotted on PVDF membranes (0.2 μm; Millipore/Immobilon) for 2 h at 100 V, 300 mA. Transfer buffer was composed of Tris-base (2.9 g/L), glycine (14.4 g/L), and MeOH (10% *v*/*v*) in dH_2_O. Unspecific binding was blocked overnight at 4 °C using 3% bovine serum albumin (BSA; Sigma-Aldrich) in Tris-buffered saline containing 0.1% Tween. Incubation with primary antibodies was conducted at 4 °C overnight followed by incubation with secondary HRP-linked anti-mouse (1:2000; Cell Signaling), anti-rabbit (1:2000; Cell Signaling), or anti-goat (1:2000; Thermo Fisher Scientific) antibodies for 45 min at RT. Secondary HRP-linked antibodies were detected using ECL Western blotting detection reagents (GE Healthcare) and a LAS-3000 luminescent image analyzer (Fujifilm). The following primary antibodies were used: rabbit anti-GFAP (1:250; Dako) rabbit anti-vimentin (1:2000; Abacm), mouse anti-nestin (1:1500; BD Biosciences), rabbit anti-GLT-1 (1:250; Novus Biological), mouse anti-βIII-tubulin (1:1500; Covance), goat anti-SOX2 (1:200; Santa Cruz), HRP-linked anti-GAPDH (1:500; Cell Signaling), and HRP-linked anti-beta-actin (1:1500; Cell Signaling). The intensity of bands was quantified using ImageJ software and normalized to GAPDH protein levels.

### BrdU Injections

For basal cell proliferation, 3-month-old male mice received a single intraperitoneal injection of BrdU (300 mg/kg) in sterile saline and were killed 24 h later. For cell fate determination, 3-month-old male mice received BrdU (300 mg/kg) injection twice daily for 1 week and were killed 6 weeks after the first injection.

### Tissue Processing and Immunohistochemistry

Mice were anesthetized and perfused by 4% PFA transcardially, and the dissected brains were postfixed with 4% PFA overnight. After immersion in 30% sucrose in PBS, 30-μm-thick coronal sections were cut on a cryostat microtome. For BrdU immunohistochemistry, sections were treated first with 2 M HCl at 37 °C for 15 min, and then blocked in 2% BSA (Sigma-Aldrich) in DPBS supplemented with 1% Triton-X 100 (Sigma-Aldrich) at 4 °C overnight. The sections were then incubated with primary antibodies at 4 °C overnight. The following antibodies were used: rat anti-BrdU (1:200; Serotec), goat anti-doublecortin (1:50; Santa Cruz), and mouse anti-NeuN (1:200; Millipore). Fluorophore-conjugated secondary antibodies were used at a dilution of 1:1000 and incubated with the sections for 1.5 h at RT. Secondary antibodies used were (all from Thermo Fisher Scientific) Alexa 594-conjugated donkey anti-rat, Alexa 488-conjugated donkey anti-goat, Alexa 488-conjugated donkey anti-rat, and Alexa 594-conjugated donkey anti-mouse. Cell nuclei were visualized by DAPI (Sigma-Aldrich).

### Hippocampal Cell Quantification

For absolute BrdU cell quantification and colocalization of BrdU with doublecortin (DCX) or NeuN, every 6th coronal section covering the dentate gyrus within the hippocampal formation (total ten sections covering a depth of 1800 μm) were used for immunohistochemical analysis. The sections were imaged by a LSM 700 confocal microscope (Zeiss) using a tiled scan function with Z-stack optical dissections covering 25 μm thickness, to capture the whole dentate gyrus area in each section. The images were analyzed by Zen 2.1 software (Zeiss). On each section, all cells positive for BrdU within the region of interest were manually quantified and assessed for NeuN or DCX immunoreactivity.

### Data Analysis

Statistical analyses were performed using either Excel (Microsoft) or GraphPad Prism (Graphpad software). Two-tailed *t* test was used for comparison between two groups. Two-way ANOVA followed by post hoc analysis (Sidak correction) was conducted for multiple comparison with repeated measurements. Difference were considered significant at *p* < 0.05. All values were presented as mean ± SEM.

## Results

### *VIM*^*SA/SA*^ Astrocytes Exhibit Well-Developed Intermediate Filament Network, Normal Proliferation, and Scratch Wound Response Despite Lower Vimentin Levels

Astrocytes lacking vimentin exhibit intermediate filaments forming tightly packed bundles that are composed of only GFAP [8], altered cell morphology, and reduced motility [46]. Thus, we first examined the intermediate filament system and morphology of primary astrocytes isolated from the cortex of 2-day-old *VIM* ^*WT/WT*^ and *VIM* ^*SA/SA*^ mice. Immunolabeling of GFAP, nestin, and vimentin showed bundles of intermediate filaments that did not differ between *VIM* ^*WT/WT*^ and *VIM* ^*SA/SA*^ astrocytes (Fig. 1a). Combined immunolabeling of *VIM* ^*WT/WT*^ and *VIM* ^*SA/SA*^ astrocytes with antibodies against GFAP and vimentin, or nestin and vimentin, visualized comparable networks of intermediate filament bundles with a comparable contribution of the respective intermediate filament proteins (Fig. 1a). Western blot analysis showed lower levels of vimentin and higher levels of GFAP in *VIM* ^*SA/SA*^ astrocytes, while the levels of nestin and glutamate transporter GLT-1 were unaltered (Fig. 1b). *VIM* ^*SA/SA*^ and *VIM* ^*WT/WT*^ astrocytes were of a comparable cell size (Fig. 1c). As *VIM* ^*SA/SA*^ fibroblasts exhibit problems with cell division [43], we next determined the fraction of astrocytes with more than one nucleus. Polynucleation was rarely observed in cultures of *VIM* ^*WT/WT*^ and *VIM* ^*SA/SA*^ astrocytes, and there was no difference in the percentage of polynucleated cells in *VIM* ^*WT/WT*^ and *VIM* ^*SA/SA*^ astrocyte cultures (1.4 ± 0.1% versus 1.3 ± 0.2%, respectively; Fig. 1c). There was no difference in the proliferation and saturation cell density of *VIM* ^*WT/WT*^ and *VIM* ^*SA/SA*^ primary astrocytes, albeit the *VIM* ^*SA/SA*^ astrocytes exhibited a trend towards increased proliferation and higher saturation cell density (Fig. 1d).

**Figure:**
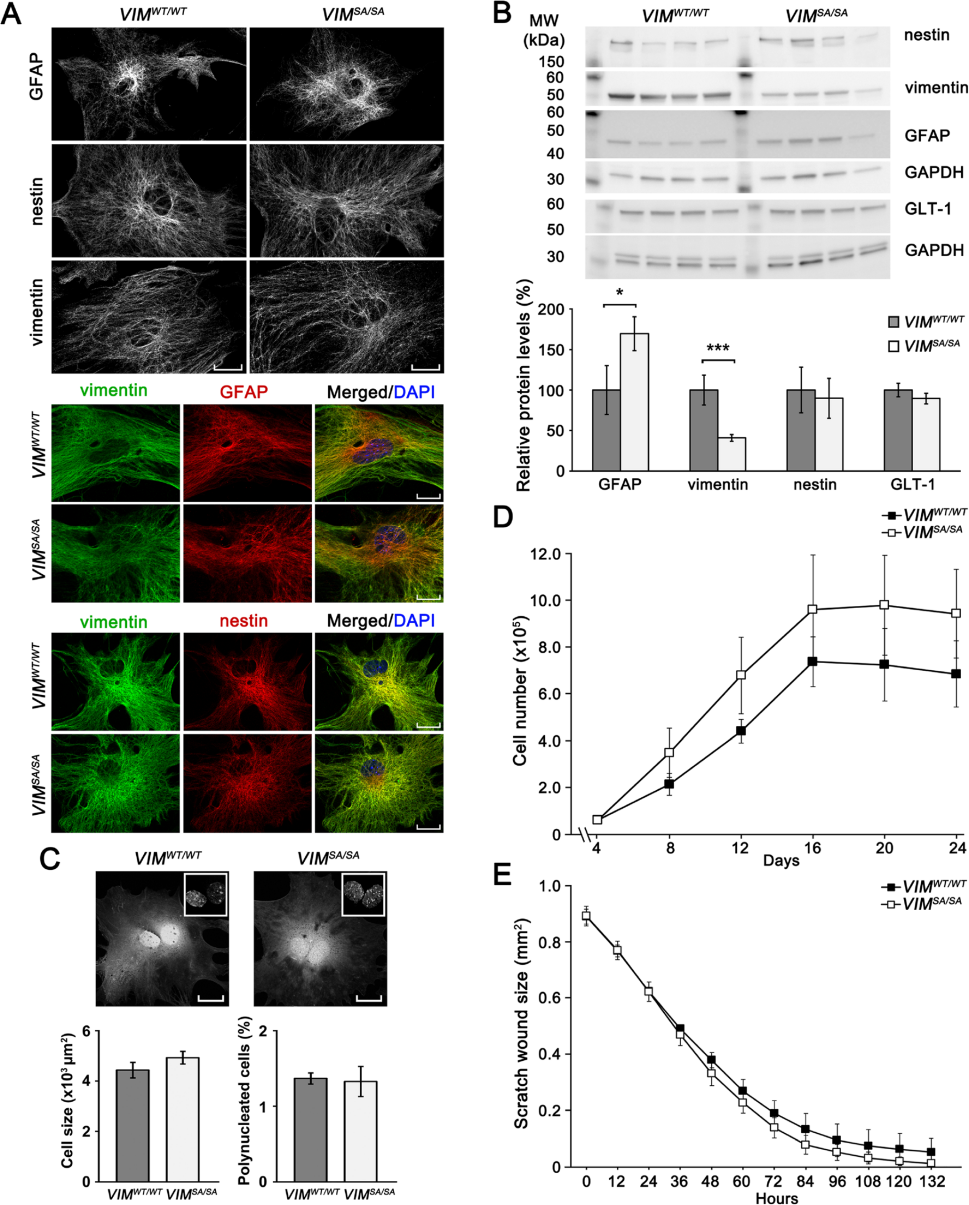

Next, we used the scratch wound assay to assess astrocyte motility. Recordings of wound closure dynamics by live cell imaging every 12 h for 132 h did not show any difference in the wound closure between *VIM* ^*WT/WT*^ and *VIM* ^*SA/SA*^ astrocytes. These results demonstrate normal motility of *VIM* ^*SA/SA*^ astrocytes (Fig. 1e).

### *VIM*^*SA/SA*^ Neurosphere Cells Show Increased Neuronal Differentiation

**Fig. 2:**
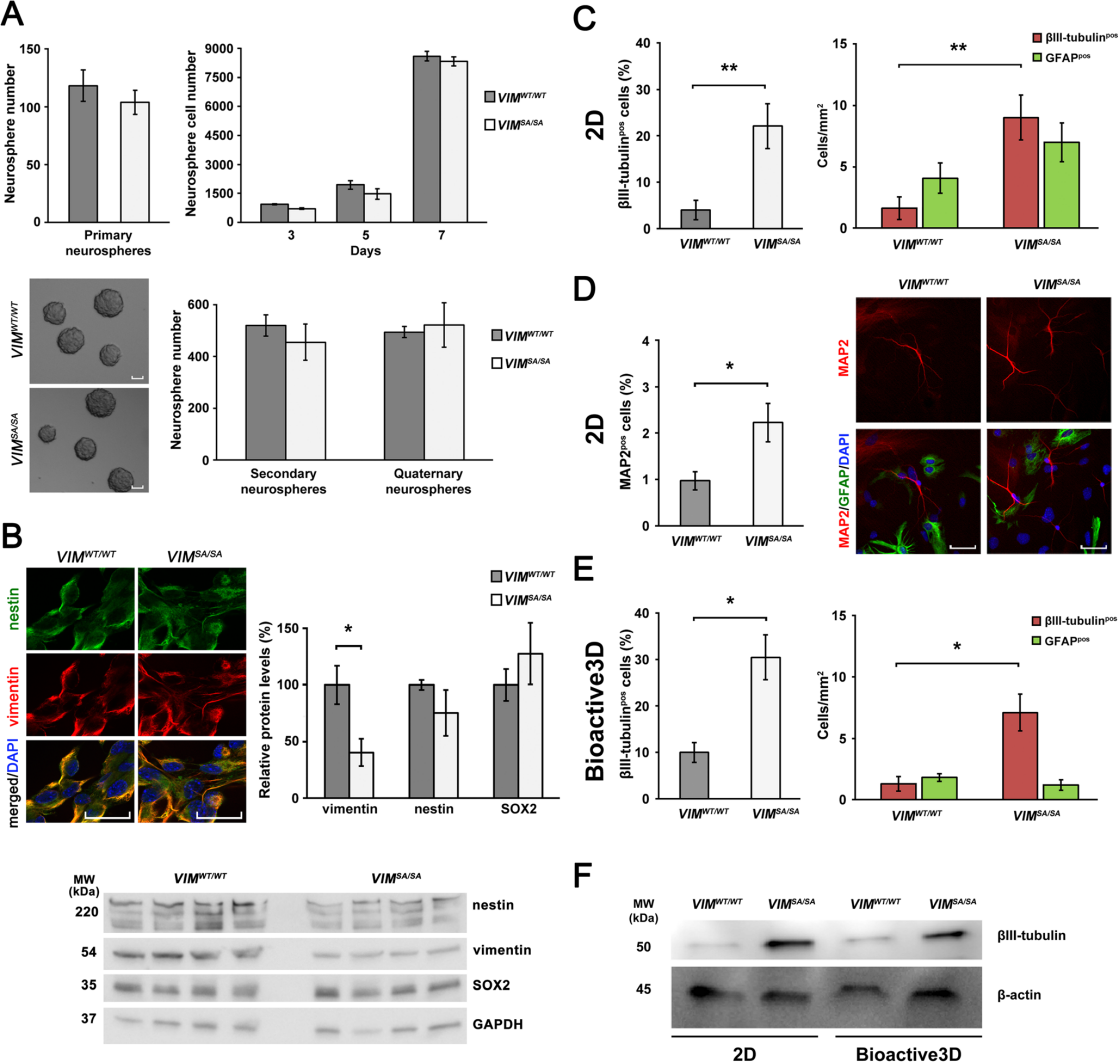
*VIM* ^*SA/SA*^ neurosphere cells show increased neuronal differentiation. **a** *VIM* ^*SA/SA*^ and *VIM* ^*WT/WT*^ brain cells showed comparable neurosphere forming capacity (assessed for primary, secondary, and quaternary neurospheres) and *VIM* ^*SA/SA*^ and *VIM* ^*WT/WT*^ neurosphere cells showed comparable proliferation. The data show the total number of neurospheres formed from 50,000 plated cells (*n* = 4 per genotype) and the number of neurosphere cells generated from primary dissociated neurospheres (*n* = 4 per genotype). **b** *VIM* ^*SA/SA*^ and *VIM* ^*WT/WT*^ neurosphere cells showed comparable vimentin and nestin immunoreactivity. Western blot analysis showed comparable expression of nestin and SOX-2 but lower expression of vimentin in *VIM* ^*SA/SA*^ neurosphere cells compared with *VIM* ^*WT/WT*^ (*n* = 4 per genotype). **c**–**e** To assess the neuronal differentiation, dissociated neurosphere cells were allowed to differentiate, and double-immunolabeled with neuronal makers βIII-tubulin or MAP2, and astrocyte marker GFAP. *VIM* ^*SA/SA*^ neurosphere cells showed highly increased relative (*left*) and absolute (*right*) neuronal differentiation compared with *VIM* ^*WT/WT*^ neurosphere cells in both 2D and Bioactive3D culture system (*n* = 5 per genotype in 2D system, *n* = 4 per genotype in Bioactive3D system; **c**, **e**, respectively). *VIM* ^*SA/SA*^ neurosphere cells showed also twofold increase of neuronal differentiation in 2D culture system when the neurons were immunolabeled with antibody against MAP2 (**d)**. **f** Western blot indicates higher expression level of βIII-tubulin in differentiated *VIM* ^*SA/SA*^ neurosphere cells than in differentiated *VIM* ^*WT/WT*^ neurosphere cells, in both 2D and 3D culture systems (*n* = 1 per genotype, pooled lysates from four samples). Data are presented as a mean ± SEM. *p < 0.05; **p < 0.01. *Scale bar* in (**a**) and (**d**), 50 μm. *Scale bar* in (**b**), 20 μm

Combined absence of GFAP and vimentin resulting in the absence of astrocyte cytoplasmic intermediate filament system was previously linked to increased neuronal differentiation of neurosphere cells [16] and increased hippocampal neurogenesis both in health and disease [16, 17, 47]. To determine the effects of the absence of mitotic phosphorylation sites in vimentin on neurosphere formation and differentiation, we assessed proliferation and differentiation of *VIM* ^*SA/SA*^ and *VIM* ^*WT/WT*^ neurosphere cells. A comparable number of primary, secondary, and quaternary neurospheres formed from the brain tissue of *VIM* ^*SA/SA*^ and *VIM* ^*WT/WT*^ mice, and the cells of dissociated *VIM* ^*SA/SA*^ and *VIM* ^*WT/WT*^ neurospheres exhibited comparable proliferation (Fig. 2a). As demonstrated by immunolabeling with antibodies against nestin and vimentin, *VIM* ^*SA/SA*^ and *VIM* ^*WT/WT*^ neurosphere cells exhibited similar nestin and vimentin immunoreactivity (Fig. 2b). However, Western blot analysis showed lower levels of vimentin in *VIM* ^*SA/SA*^ neurosphere cells compared with *VIM* ^*WT/WT*^ and comparable levels of neural stem cell markers nestin and SOX2 (Fig. 2b).

Next, we assessed the differentiation of *VIM* ^*SA/SA*^ and *VIM* ^*WT/WT*^ neurosphere cells, both in a standard two-dimentional (2D) cell culture system and in the 3D cell culture system (Bioactive3D) that we previously developed for astrocytes and neurons [45, 48–50]. When allowed to differentiate in a 2D cell culture system, the dissociated neurosphere cells derived from *VIM* ^*SA/SA*^ mice were > 4 times more likely to differentiate into βIII-tubulin^pos^GFAP^neg^ neurons than neurosphere cells derived from *VIM* ^*WT/WT*^ mice (22.1 ± 4.8% versus 4.1 ± 2.1%, respectively; Fig. 2c). The *VIM* ^*SA/SA*^ and *VIM* ^*WT/WT*^ neurosphere cells exhibited comparable astrocyte differentiation (Fig. 2c). The increased neuronal differentiation of *VIM* ^*SA/SA*^ neurosphere cells was confirmed by immunostaining with antibodies against MAP2, a marker of more mature neurons compared with those visualized by antibodies against βIII-tubulin. We observed more than 100% increase in the fraction of MAP2 positive cells in *VIM* ^*SA/SA*^ compared with *VIM* ^*WT/WT*^ neurosphere cell cultures (Fig. 2d). *VIM* ^*SA/SA*^ neurosphere cells differentiating in the Bioactive3D cell culture system generated less astrocytes than in the 2D system (Fig. 2c, e). Similar to the 2D culture system, in Bioactive3D, neurosphere cells derived from *VIM* ^*SA/SA*^ mice were > 3 times more likely to differentiate into βIII-tubulin^pos^GFAP^neg^ neurons than neurosphere cells derived from *VIM* ^*WT/WT*^ mice (30.4 ± 6.9% versus 10.0 ± 4.4%, respectively; Fig. 2e), while *VIM* ^*SA/SA*^ and *VIM* ^*WT/WT*^ neurosphere cells exhibited comparable astrocyte differentiation (Fig. 2e). The increased neuronal differentiation potential of *VIM* ^*SA/SA*^ neurosphere cells in both culture systems was further supported by the βIII-tubulin levels on Western blot in pooled protein lysates from cultures of differentiating *VIM* ^*SA/SA*^ neurosphere cells compared with *VIM* ^*WT/WT*^ (Fig. 2f).

### The Pro-neurogenic Properties of *VIM*^*SA/SA*^ Neurosphere Cells Are Not Affected by the Astrocyte Environment

**Fig. 3:**
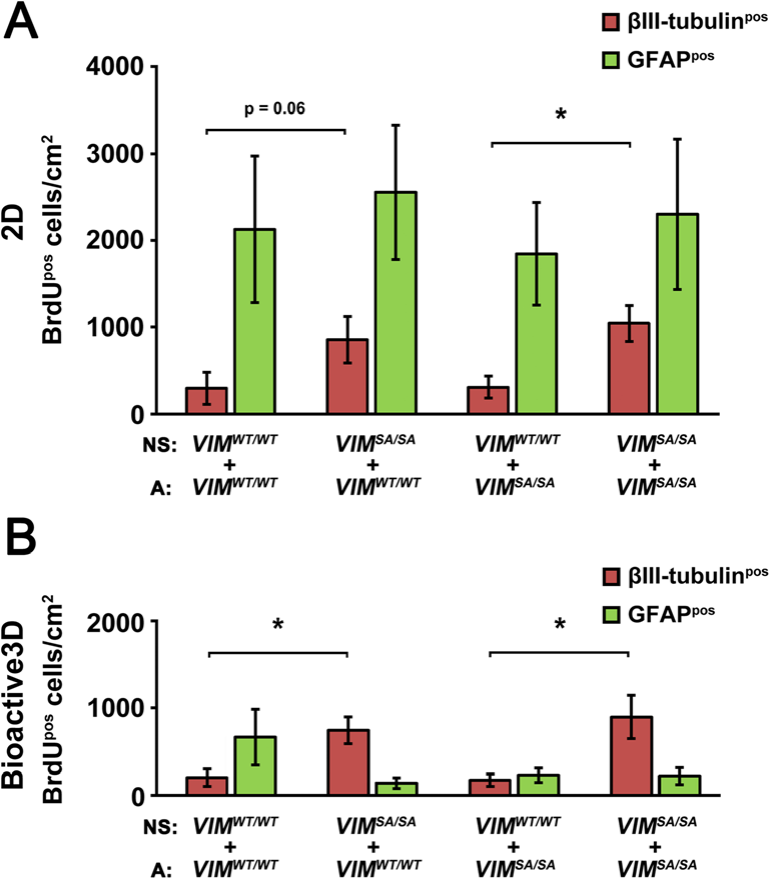
The pro-neurogenic properties of *VIM* ^*SA/SA*^ neurosphere cells are not affected by the astrocyte environment. **a**, **b** Dissociated *VIM* ^*WT/WT*^ or *VIM* ^*SA/SA*^ neurosphere cells pre-labeled with BrdU cocultured with *VIM* ^*WT/WT*^ or *VIM* ^*SA/SA*^ astrocytes in 2D (**a**) and Bioactive3D (**b**) culture systems. The graphs show the absolute number of immunolabeled neurons (βIII-tubulin^pos^/GFAP^neg^/BrdU^pos^) and astrocytes (GFAP^pos^/βIII-tubulin^neg^/BrdU^pos^) per square millimeter (*n* = 5 per genotype in 2D cultures, *n* = 4 per genotype in Bioactive3D cultures; **a**, **b**, respectively). Data are presented as a mean ± SEM. *p < 0.05. *NS*, neurosphere cells; *A*, astrocytes

We previously reported that reduced Notch signaling from intermediate filament-free astrocytes increased neuronal differentiation of neural progenitor cells [16, 21]. Therefore, we next sought to determine whether the increase in neuronal differentiation of *VIM* ^*SA/SA*^ neurosphere cells was neurosphere cell intrinsic or caused by the niche of *VIM* ^*SA/SA*^ astrocytes. We assessed neurogenesis and astrogenesis from *VIM* ^*SA/SA*^ and *VIM* ^*WT/WT*^ neurosphere cells in cocultures of primary neurospheres, pre-labeled with BrdU for 48 h, and passage 1 astrocytes. In the 2D cell culture system, *VIM* ^*SA/SA*^ neurosphere cells gave rise to more neurons than *VIM* ^*WT/WT*^ neurosphere cells when cocultured with *VIM* ^*SA/SA*^ astrocytes (10.4 ± 2.1 cells/mm^2^ versus 3.1 ± 1.3 cells/mm^2^, respectively; Fig. 3a). Similarly, in the Bioactive3D cell culture system, *VIM* ^*SA/SA*^ neurosphere cells gave rise to more neurons than *VIM* ^*WT/WT*^ neurosphere cells, irrespective of whether they were cocultured with *VIM* ^*WT/WT*^ (7.4 ± 1.5 cells/mm^2^ versus 2.0 ± 1.0 cells/mm^2^, respectively) or *VIM* ^*SA/SA*^ astrocytes (9.0 ± 2.5 cells/mm^2^ versus 1.7 ± 0.7 cells/mm^2^, respectively; Fig. 3b). The astrocyte environment did not affect astrogenesis from neurosphere cells in any of the coculture systems (Fig. 3a, b). These results suggest that the increased neuronal differentiation of *VIM* ^*SA/SA*^ neurosphere cells is a cell intrinsic phenomenon.

### *VIM*^*SA/SA*^ Mice Exhibit a Modest Increase in the Fraction of Newly Born and Surviving Neurons in the Hippocampal Dentate Gyrus

**Fig. 4:**
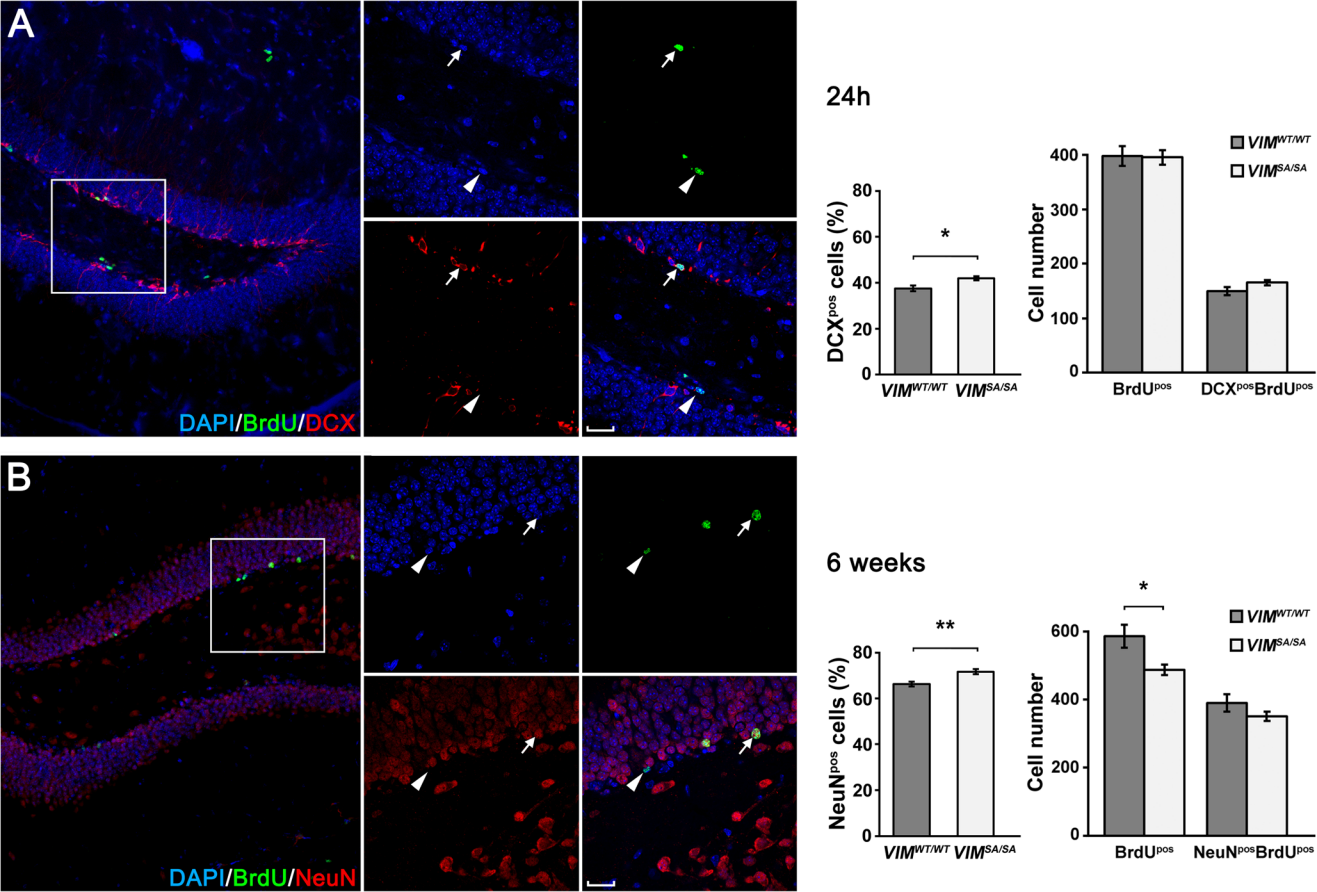
*VIM* ^*SA/SA*^ mice exhibit a modest increase in the fraction of newly born and surviving neurons in the hippocampal dentate gyrus. **a** Image of dentate gyrus immunostained with antibodies against doublecortin (*DCX*) and BrdU; the nuclei were visualized with DAPI. *Arrow*, DCX^pos^BrdU^pos^ cell; *arrowhead*, DCX^neg^BrdU^pos^ cell. *VIM* ^*SA/SA*^ mice had higher percentage of DCX^pos^BrdU^pos^ neuroblasts among the proliferation cells (BrdU^pos^) in the dentate gyrus, but the absolute numbers of BrdU^pos^ cells and DCX^pos^BrdU^pos^ neuroblasts were comparable in *VIM* ^*WT/WT*^ and *VIM* ^*SA/SA*^ mice. Ten serial coronal brain sections with a 180-μm inter-section distance analyzed per mouse, *n* = 5 per genotype. **b** Image of dentate gyrus immunostained with antibodies against NeuN and BrdU. The nuclei were visualized with DAPI. *Arrow*, NeuN^pos^BrdU^pos^ cell; *arrowhead*, NeuN^neg^BrdU^pos^ cell. *VIM* ^*SA/SA*^ mice showed higher percentage of neurons (NeuN^pos^BrdU^pos^) among surviving newly born cells (BrdU^pos^), but the absolute number of surviving newly born neurons was comparable between the genotypes. *VIM* ^*SA/SA*^ mice showed decreased absolute number of surviving newly born cells (BrdU^pos^). Ten serial coronal brain sections with a 180 μm inter-section distance analyzed per mouse, *n* = 10 per genotype. Data are presented as a mean ± SEM. *p < 0.05; **p < 0.01. The *inlets* in (**a**) and (**b**) show individual fluorescence channels. *Scale bar* in (**a**) and (**b**), 20 μm

To ascertain whether the increased neuronal differentiation of *VIM* ^*SA/SA*^ neurosphere cells in vitro translates into increased neurogenesis in the brains of *VIM* ^*SA/SA*^ mice, we used BrdU in vivo labeling and quantified neural stem cell proliferation and neurogenesis in the hippocampal dentate gyrus, one of the two major neurogenic regions of the adult brain. Twenty-four hours after a single injection of BrdU, *VIM* ^*SA/SA*^ mice had higher percentage of DCX^pos^BrdU^pos^ neuroblasts in the dentate gyrus of the hippocampus (41.9 ± 0.9% versus 37.6 ± 1.2% of the BrdU^pos^ cells, respectively); however, the absolute numbers of BrdU^pos^ cells and DCX^pos^BrdU^pos^ neuroblasts was comparable in *VIM* ^*WT/WT*^ and *VIM* ^*SA/SA*^ mice (Fig. 4a).

Six weeks following BrdU labeling, the percentage of the newly born and surviving NeuN^pos^BrdU^pos^ neurons in *VIM* ^*SA/SA*^ mice was increased by 8% (66.3 ± 1.0% versus 71.7 ± 1.1% of the BrdU^pos^ cells; Fig. 4b). *VIM* ^*SA/SA*^ mice had lower absolute number BrdU^pos^ cells (488 ± 16 versus 586 ± 33, respectively), and the absolute numbers of newly born and surviving NeuN^pos^BrdU^pos^ neurons were comparable between the two groups (390 ± 26 versus 351 ± 14, respectively; Fig. 4b).

## Discussion

Our results show that mutation of the serine sites that are phosphorylated during mitosis in the intermediate filament protein vimentin results in lower levels of vimentin and higher levels of GFAP in *VIM* ^*SA/SA*^ astrocytes but does not affect the appearance and distribution of the intermediate filament network, astrocyte morphology, proliferation, presence of multiple cell nuclei, and astrocyte motility. Thus, phosphorylation of vimentin during mitosis does not seem to play a major role in the control of morphology, cell division, and motility of astrocytes. This is in contrast with *VIM* ^*SA/SA*^ lens epithelial cells or fibroblasts that showed disrupted intermediate filament network and formation of unbreakable intermediate filament bridges during mitosis. Lower levels of vimentin in *VIM* ^*SA/SA*^ astrocytes apparently do not affect the appearance and function of the intermediate filament network in these cells. Vimentin network is highly dynamic with an active subunit exchange between polymers and soluble subunits [51], and it is conceivable that deficit in vimentin phosphorylation affects the equilibrium between the polymerized and unpolymerized intermediate filament proteins and consequently leads to increased degradation of soluble vimentin subunits. Functionally deficient intermediate filament network in *VIM* ^*SA/SA*^ lens epithelial cells and fibroblasts was linked to the increased incidence of polynucleation observed in these cells [42, 43]. The presence of an intact intermediate filament network in *VIM* ^*SA/SA*^ astrocytes is compatible with the normal incidence of polynucleation in these cells. Vimentin is highly expressed in astrocytes, lens epithelial cells and fibroblasts. In astrocytes, vimentin forms intermediate filaments together with GFAP and nestin. Given that GFAP is highly expressed in postnatal astrocytes but not in lens epithelial cells or fibroblasts [42, 43, 52], and that GFAP expression levels were increased in *VIM* ^*SA/SA*^ astrocytes, it is possible that in *VIM* ^*SA/SA*^ astrocytes, GFAP compensates for the potential dysfunction of vimentin. Compensation by GFAP is conceivably also the explanation for the normal appearance of the intermediate filament network in *VIM* ^*SA/SA*^ astrocytes, in contrast with the prominent changes of the intermediate filament system in *VIM* ^*SA/SA*^ epithelial cells.

We previously reported that the absence of GFAP and vimentin (but not of only GFAP or of only vimentin) led to decreased Notch signaling from astrocytes to neural progenitor cells [16, 21] and increased neuronal differentiation of these cells [16] as well as increased hippocampal neurogenesis in mice devoid of astrocyte intermediate filaments, both in health and disease [16, 17, 47]. Here, we observed increased neuronal differentiation of *VIM* ^*SA/SA*^ neurosphere cells and determined that the pro-neurogenic properties of *VIM* ^*SA/SA*^ neurosphere cells were not due to astrocyte environment but were neurosphere cell intrinsic.

In both 2D and Bioactive3D cell culture systems, *VIM* ^*SA/SA*^ neurosphere cells showed highly increased neuronal differentiation (two- to fourfold), but the fraction of newly formed and surviving neurons in the hippocampal dentate gyrus of the *VIM* ^*SA/SA*^ mice was only modestly increased (by 8%). Thus, the in vitro effect of *VIM* ^*SA/SA*^ mutation can be mitigated or masked in vivo by niche-related factors that remain to be identified. It is interesting to point out that the Bioactive3D culture system, which maintains aspects of in vivo-like morphology of the cultured neural cells (astrocytes and neurons) and reduces the baseline reactivity of astrocytes as well as their proliferation [45, 48–50], led to lower numbers of astrocytes in the neurosphere cell differentiation assays, as well as in cocultures of astrocytes/neurosphere cells. Nevertheless, even in Bioactive3D, a cell culture system closer to the in vivo situation, the increased neurogenesis from neurosphere cells was very robust and more prominent compared with only modestly increased hippocampal neurogenesis in *VIM* ^*SA/SA*^ mice, conceivably due to the absence of other niche constituents such as endothelial cells and microglia in the in vitro system.

In conclusion, our main finding is that *VIM* ^*SA/SA*^ astrocytes do not show any major phenotypic changes, but *VIM* ^*SA/SA*^ neurospheres exhibit highly increased neuronal differentiation; this effect is neurosphere cell intrinsic and not dependent on the astrocyte niche. *VIM* ^*SA/SA*^ mice show a modest increase in the fraction of newly born and surviving neurons in the hippocampal dentate gyrus. The functional significance of this finding and the involvement of phosphovimentin in the functions of other cells that express vimentin in the neurogenic niche, such as the endothelial cells and microglia, warrant further investigation.
